# Supplementary material for: Utilizing Constant Energy Difference between sp-Peak and C 1s Core Level in Photoelectron Spectra for Unambiguous Identification and Quantification of Diamond Phase in Nanodiamonds
Source: Nanomaterials (Basel). 2024 Mar 27;14(7):590. doi: 10.3390/nano14070590 (PMC11013481; doi:10.3390/nano14070590)
Supplement: Supplementary file 1 [file nanomaterials-14-00590-s001.zip › nanomaterials-2927583-supplementary.pdf]

In Figure S1, images of the prepared DND-H/Au and graphitized DND-H/Au samples are shown. The as-prepared ND film was transparent before annealing, and it became dark and non-transparent after graphitization.

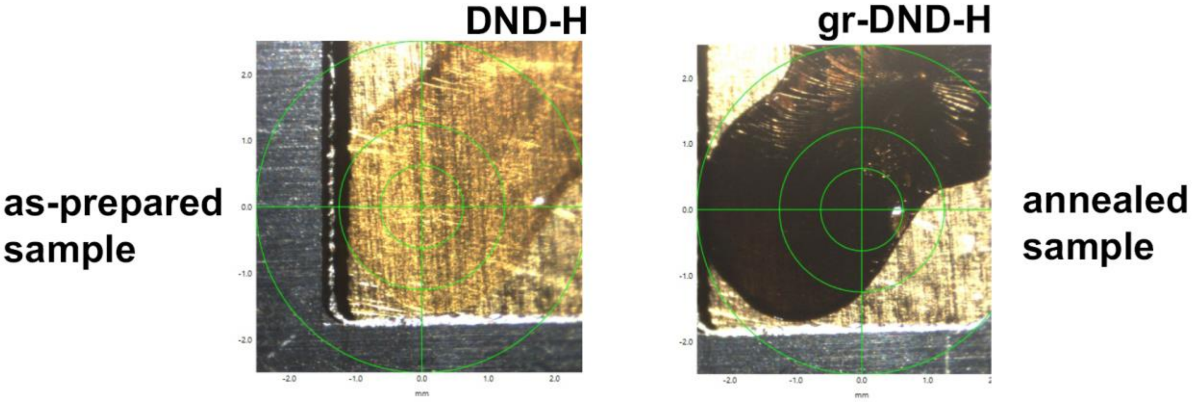

Figure S1. Picture of as-prepared and graphitized DND-H sample.

XPS survey spectra of as-prepared, annealed, and ArCIB-sputtered ND samples are shown in Figure 2. Residual oxygen contaminants were observed on the sample surfaces. The signal from the Au substrate was distinguishable only for DND-H samples. Nitrogen was detected in DND-H samples and was absent in snr-DND-H samples.

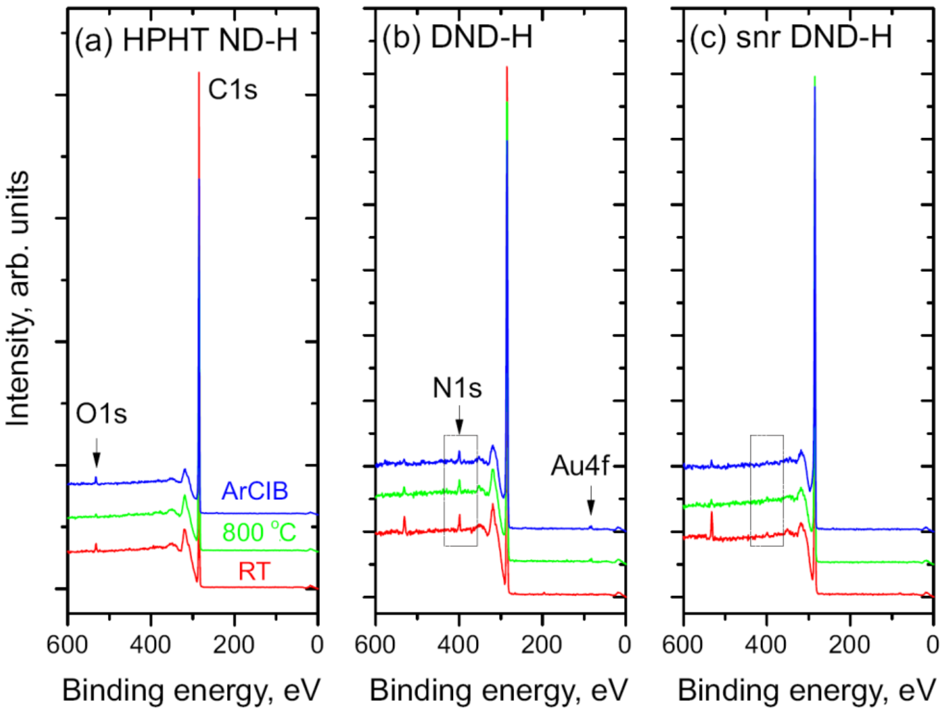

Figure S2. Survey XPS spectra from as-prepared samples measured at room temperature (red), from annealed samples in UHV at 800 °C (green), and from ArCIB sputtered samples at RT (blue).

Figure S3 shows (a) the oxygen and (b) nitrogen atomic concentrations observed during the annealing of ND samples, after cooling to room temperature, and after ArCIB sputtering. Concentrations were derived from the areas of the high-resolution core level XPS spectra. Initially, the concentration of oxygen was higher (approximately 3 at.%) for the snr-DND-H sample and lower (about 1 at.%) for the HPHT ND-H sample. After annealing

at 800 °C, oxygen concentrations dropped below 1 at.% for all samples, indicating partial oxygen desorption from the DND surfaces. The concentration of nitrogen in the DND-H sample remained nearly constant during annealing and sputtering, indicating rather incorporation of N into the ND cores. The oxygen desorption from the ND surfaces during annealing has no obvious correlation with graphitization temperature change.

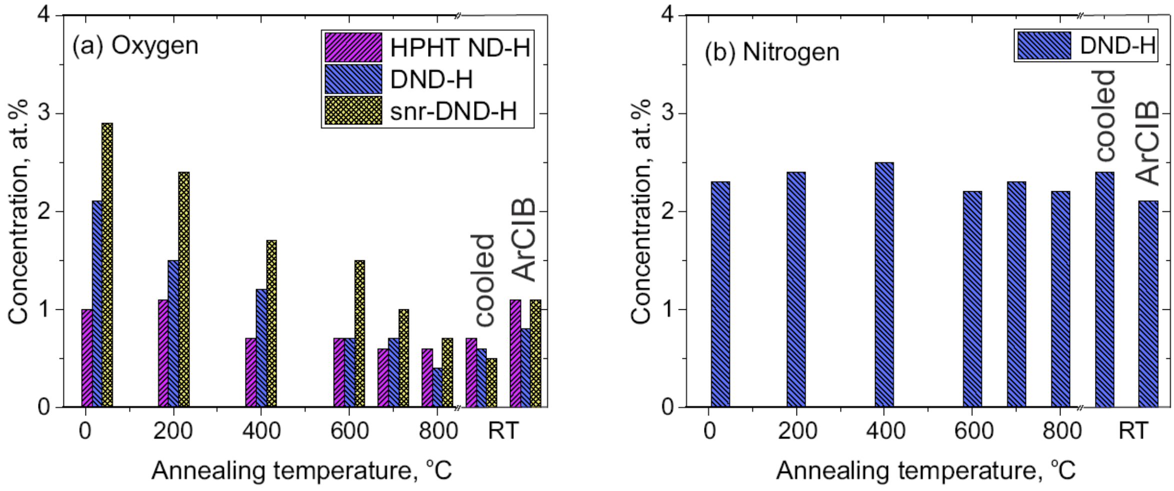

Figure S3. The measured atomic concentrations of (a) oxygen and (b) nitrogen during the annealing and sputtering. The samples were heated from room temperature (RT) to 800 °C, followed by cooled to RT, and subsequently sputtered by with argon clusters ion beam (ArCIB) at RT.

The high-resolution N 1s spectra are presented in Figure S4 (a), (b). No nitrogen signal was resolved for snr-DND-H sample. The binding energies of N 1s spectra in (a) were calibrated according to the position of the C 1s peaks at 285.0 eV (see description in the manuscript). Two components of N 1s were resolved [1,2] at 399.1 eV (C-N, C-NH bonds) and at 402.5 eV, which is probably related to the (ammonium) nitrites [3], i.e., it could involve the C-(N,O) group of bonds. Recently, the formation of nitrate ions was observed during DND sonication [4]. It should be mentioned that annealing and sputtering of NDs reduced the oxygen concentration, but no significant change in N2/N1 peak ratios was resolved during annealing and cooling [Figure S4 (c)].

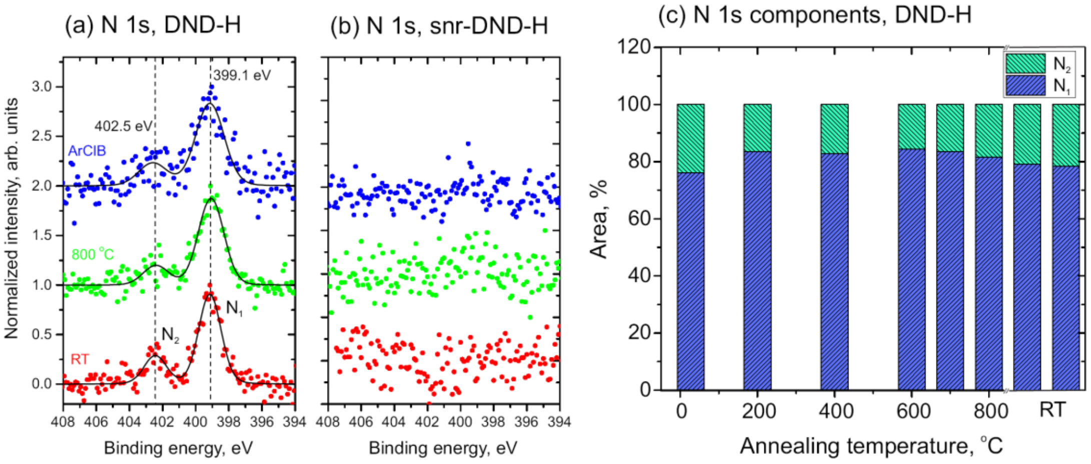

Figure S4. N 1s core level spectra obtained from (a) DND-H and (b) snr-DND-H samples at RT, 800 °C and after ArCIB sputtering. No nitrogen was detected in the snr-DND-H sample. (c) The dependence of N 1s component areas on annealing temperature and ArCIB sputtering.

Core level shifts (CLS) in the C 1s spectra are summarized in Table SM1 for reference bulk samples and ND samples. The reference samples were measured at room temperature (RT), while the ND samples underwent annealing and sputtering at RT.

Table S1. Core level shifts in eV obtained by deconvolution of C 1s peaks.

		CLS in resp. 285 eV				
		Components				
Sample	treatment	sp^3	sp^2	Cx/C-N	C-O/C-OH	C=O
C(111)-H	RT	0.0	-0.8	0.5	1.0-1.5	-
HOPG	RT	-	-0.6	-	-	-
HPHT ND-H	RT	0.0	-0.9	0.8	1.7	-
	800 °C	0.0	-1.0	0.8	1.7	-
	ArCIB, RT	0.0	-0.8	0.8	1.7	-
DND-H	RT	0.0	-0.9	0.8	1.2	2.3
	800 °C	0.0	-0.9	0.8	1.2	2.3
	ArCIB, RT	0.0	-1.5	0.8	1.5	2.2
snr-DND-H	RT	0.0	-0.9	0.8	1.2	2.2
	800 °C	0.0	-0.9	0.8	1.2	2.2
	ArCIB, RT	0.0	-0.9	0.8	1.2	2.2

The surface morphology of the ND samples was analysed using field-emission scanning electron microscopy (FE-SEM, Tescan MAIA3) with an electron beam energy of 10 keV. SEM images of the as-prepared RT samples and the ArCIB-sputtered samples (inside craters) are presented in Figure S5. Upon drop-casting and drying on Au substrates, large agglomerates of diamond NPs were formed. Figure S5 (a) reveals diamond grains in the tens of nanometers in size, while Figure S5 (b) displays a porous film. For the snr-DND-H sample, NPs aggregated into large clusters on the surface, making it impossible to distinguish individual grains.

After ArCIB sputtering, the HPHT ND-H surface became coated with a graphitic film, as indicated by the black patterns in Figure S5 (d), and no grains were discernible. This observation is consistent with patterns seen in SEM images of sputtered nanocrystalline diamond films (NCD) [5]. In contrast, the DND-H sample showed no significant change in morphology of sputtered samples [as depicted in Fig. 6 (e)].

As for the snr-DND-H sample, ArCIB sputtering led to a flattening of the ND cluster surface, possibly due to the surface being polished by the Ar cluster ion beam.

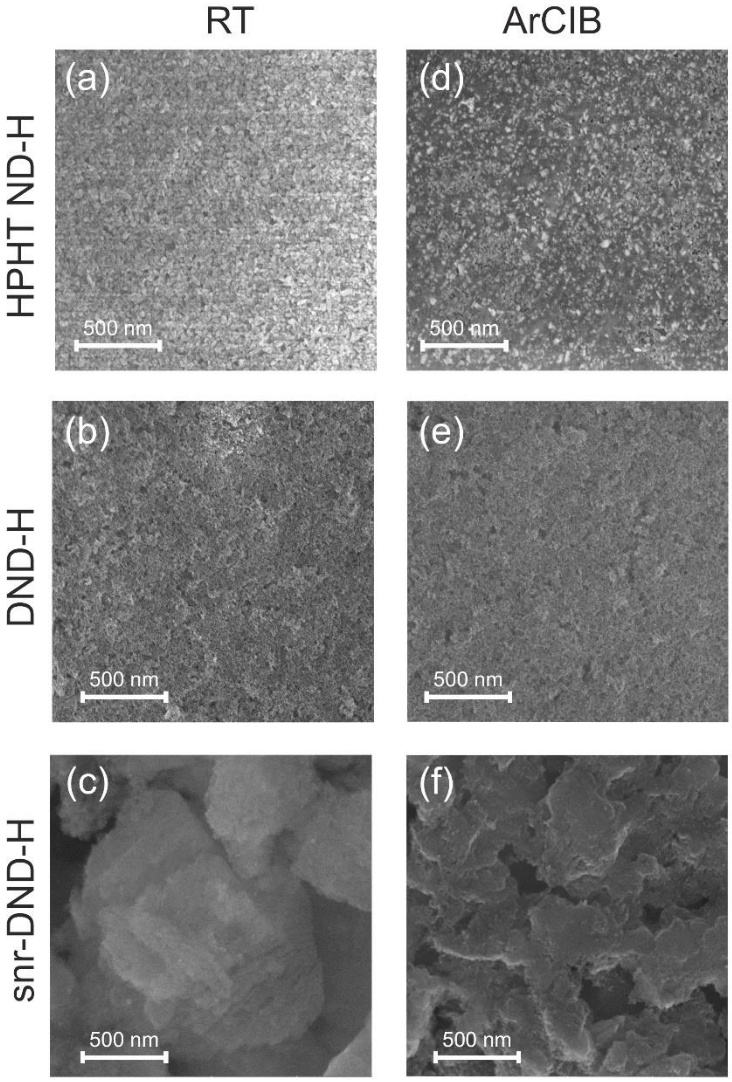

Figure S5. SEM images of as-prepared (a-c) ND samples measured at RT, and (d-f) their ArCIB sputtered counterparts measured at the bottom of the craters. Sputtered surfaces became smoother and were covered by graphitic carbon layers [black patterns, well-resolved in (d)].

Figure S6 shows Raman spectra of as-prepared ND samples measured by UV laser (325 nm excitation).

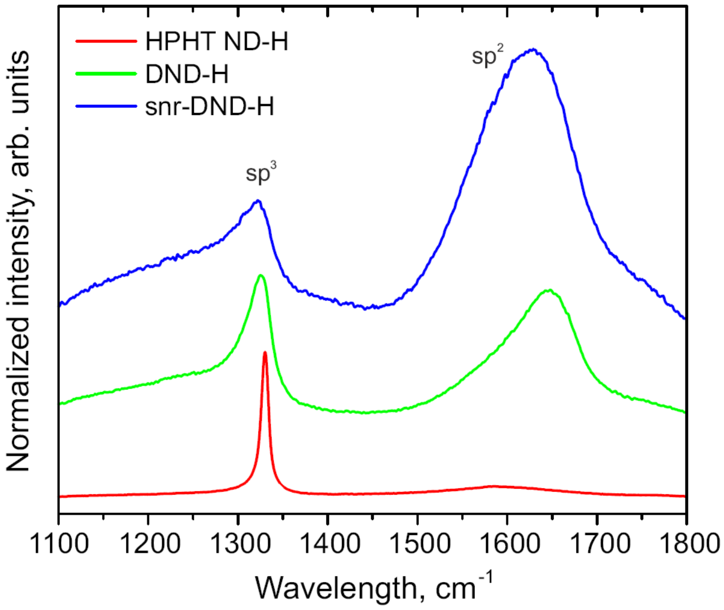

Figure S6. UV-Raman spectra of as-prepared ND samples sp3 peak intensity decreases with NP size decrease, whereas the sp2 peak intensity increases.

References

1. S. Stehlik, M. Mermoux, B. Schummer, O. Vanek, K. Kolarova, P. Stenclova, A. Vlk, M. Ledinsky, R. Pfeifer, O. Romanyuk, I. Gordeev, F. Roussel-Dherbey, Z. Nemeckova, J. Henych, P. Bezdicka, A. Kromka, B. Rezek, Size Effects on Surface Chemistry and Raman Spectra of Sub-5 nm Oxidized High-Pressure High-Temperature and Detonation Nanodiamonds, J. Phys. Chem. C. 125 (2021) 5647–5669. https://doi.org/10.1021/acs.jpcc.0c09190.
2. V.R. Dhanak, Yu.V. Butenko, A.C. Brieva, P.R. Coxon, L. Alves, L. Šiller, Chemical Functionalization of Nanodiamond by Amino Groups: An X-Ray Photoelectron Spectroscopy Study, J. Nanosci. Nanotech. 12 (2012) 3084–3090. https://doi.org/10.1166/jnn.2012.4547.
3. J.F. Moulder, J. Chastain, eds., Handbook of X-ray photoelectron spectroscopy: a reference book of standard spectra for identification and interpretation of XPS data, Update, Perkin-Elmer Corporation, Eden Prairie, Minn, 1992.
4. F. Ducrozet, H.A. Girard, T. Jianu, S. Peulon, E. Brun, C. Sicard-Roselli, J.-C. Arnault, Unintentional formation of nitrate and nitrite ions during nanodiamonds sonication: A source of radical and electron scavengers, Colloids and Surfaces A: Physicochemical and Engineering Aspects. 663 (2023) 131087. https://doi.org/10.1016/j.colsurfa.2023.131087.
5. O. Romanyuk, J. Zemek, J. Houdková, O. Babčenko, E. Shagieva, K. Beranová, A. Kromka, P. Jiříček, Effects of monoatomic and cluster bombardment with Ar ion beam on the surface of hydrogenated nanocrystalline diamond, Diamond and Related Materials. 133 (2023) 109748. https://doi.org/10.1016/j.diamond.2023.109748.
